# Supplementary material for: A methodology for the design of experiments in computational intelligence with multiple regression models
Source: PeerJ. 2016 Dec 1;4:e2721. doi: 10.7717/peerj.2721 (PMC5136129; doi:10.7717/peerj.2721)
Supplement: Data S1 — Datailed results from UC Irvine Machine Learning Repository (Housing, Machine CPU, Wine Quality, Automobile and Parkinson) and the 3 Use Cases (Protein Corona, Gajewicz Metal Oxides and Aquatic Toxicity) [file peerj-04-2721-s001.zip › 6_UseCases/1_ProteinCorona/SVMRFE.details.csv.repeatedcv.split2.pdf]

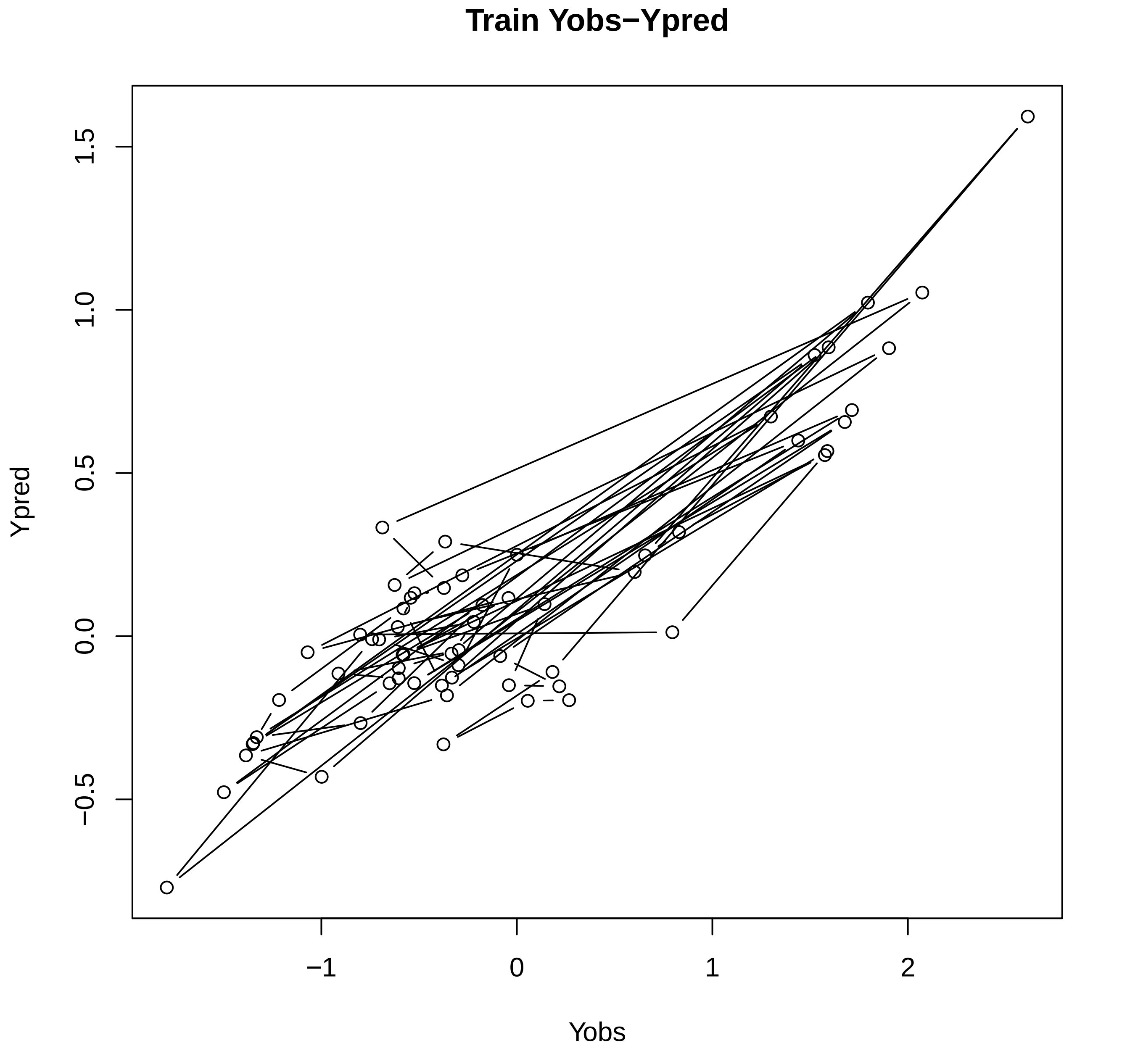

Train Yobs−Ypred
Ypred
Yobs
1.5
1.0
0.5
0.0
−0.5
−1
0
1
2

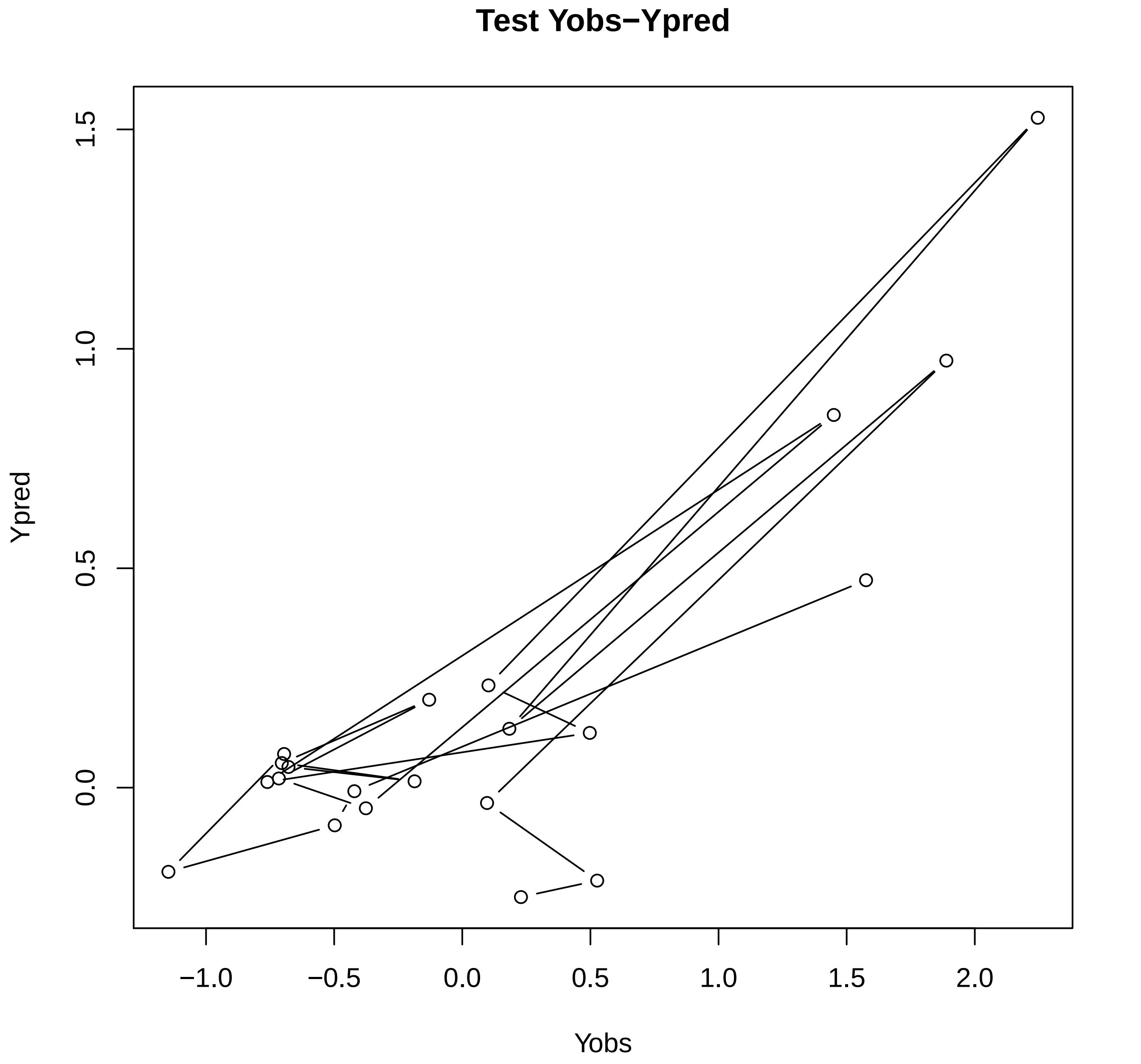
Test Yobs−Ypred
Ypred
Yobs
0.0
0.5
1.0
1.5
−1.0
−0.5
0.0
0.5
1.0
1.5
2.0

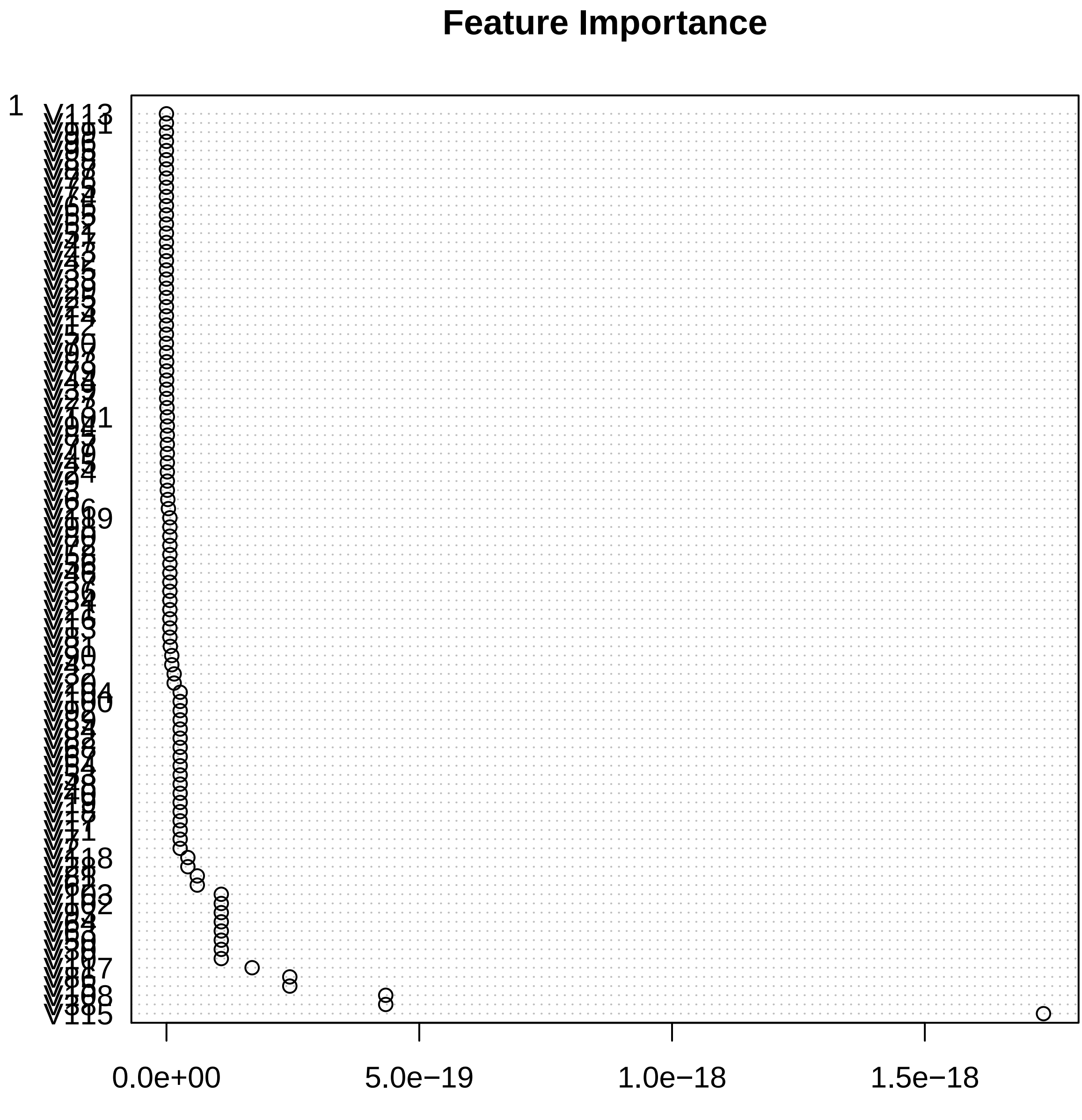

Feature Importance
0.0e+00
5.0e−19
1.0e−18
1.5e−18
V113
V115

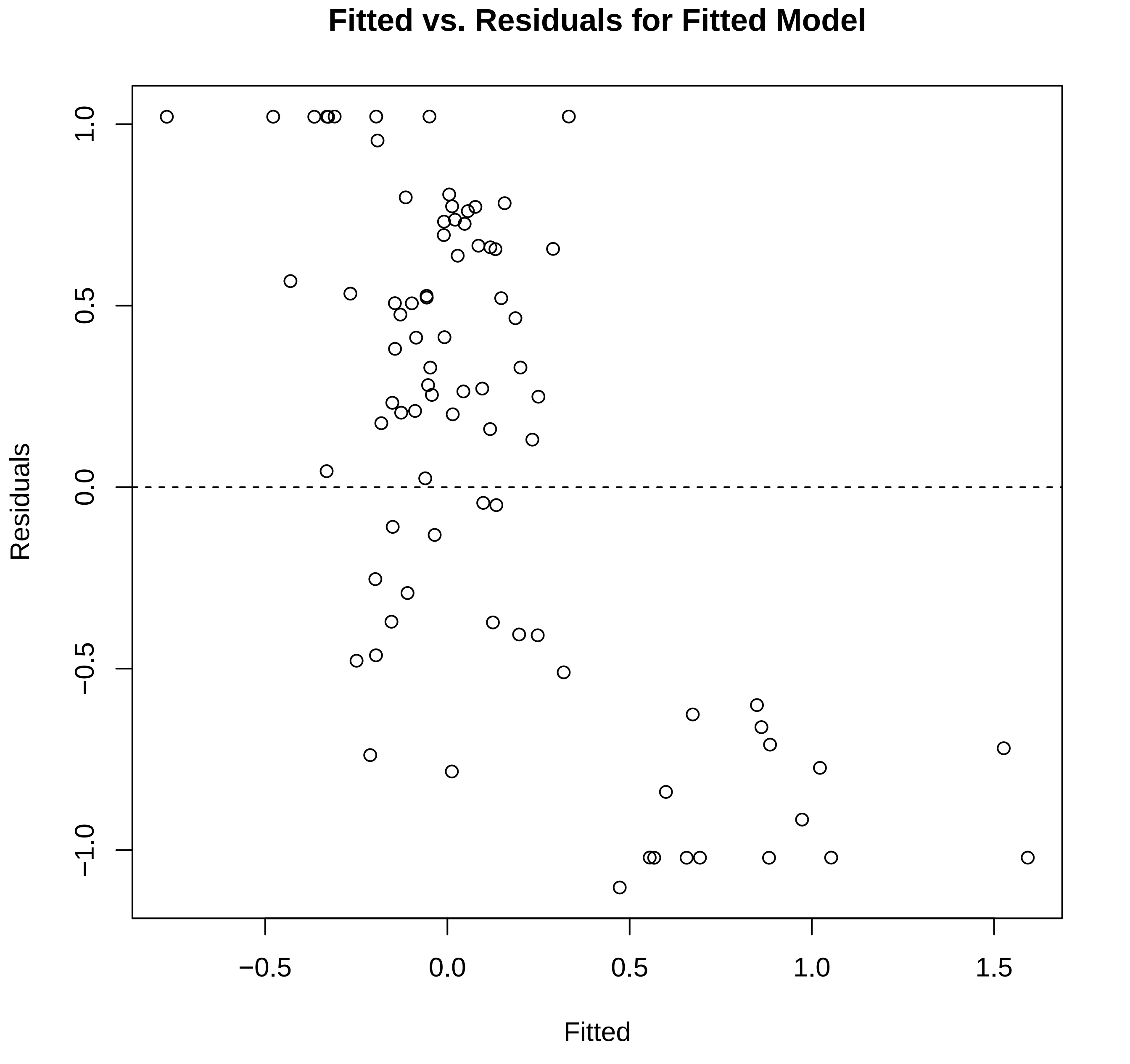

Fitted vs. Residuals for Fitted Model
Residuals
Fitted
1.0
0.5
0.0
−0.5
−1.0
−0.5
0.0
0.5
1.0
1.5
